# Clinical predictors of intracranial injuries on CT in infants younger than 2 years old with mild traumatic brain injury

**DOI:** 10.18632/oncotarget.21512

**Published:** 2017-10-04

**Authors:** Guangfu Di, Hua Liu, Xinhua Hu, Sansong Chen, Zhichun Wang, Hongyi Liu

**Affiliations:** ^1^ Department of Neurosurgery, The Affiliated Brain Hospital, Nanjing Medical University, Nanjing, China; ^2^ Department of Neurosurgery, Yijishan Hospital, Wannan Medical College, Wuhu City, China; ^3^ Department of Neurosurgery, The First People's Hospital of Kunshan, Jiangsu University, Suzhou, China

**Keywords:** intracranial injury, mild traumatic brain injury, infants

## Abstract

**Purpose:**

Mild traumatic brain injury (TBI) is common in children. The aim of this study was to identify clinical predictors of intracranial injuries on computed tomography (CT) in infants younger than 2 years old with mild TBI, which allow reducing number of imaging.

**Results:**

Of 214 enrolled infants with complete data, 30 (5.8%) sustained intracranial injuries. Younger age in months, severe injury mechanism and scalp hematomas were associated with traumatic intracranial injuries on CT. 71 had scalp hematomas and 143 had no scalp hematoma. Within infants with scalp hematomas, 26 sustained intracranial injuries and 45 presented normal. Intracranial injuries were significantly correlated with larger scalp hematomas and different scalp hematoma locations. Logistic regression analysis showed that scalp hematoma and mechanism of injury in infants younger than 2 years old with mild TBI was related to intracranial injuries (hazard ratio=38.291, P=0.0001; hazard ratio=0.174, P=0.001). In subgroup of mild TBI infants with scalp hematomas, logistic regression analysis showed age, scalp hematoma size and mechanism of injury were independently associated with intracranial injuries (hazard ratio=0.299, P=0.032; hazard ratio=5.272, P=0.006; hazard ratio=0.312, P=0.030).

**Methods:**

Between 2014 and 2016, we retrospectively enrolled infants <2 years old with mild TBI. Data recorded included age, sex, mechanism of head injury, size and location of scalp hematoma, fracture and intracranial injuries on CT.

**Conclusion:**

The characteristics of scalp hematomas and mechanism of injury were associated with intracranial injuries. These factors should be considered when making decisions on radiologic examinations of infants < 2 years old with mild TBI and alternative procedures, which do not involve ionizing radiation, should be used if appropriate.

## INTRODUCTION

Traumatic brain injury (TBI) remains the important cause of both morbidity and mortality in children. With more than 700,000 reported pediatric TBI occurring annually, and many more unreported, this represents a major pediatric health problem [1, 2]. Fortunately, most TBI in children are classified as mild TBI, require no specific therapy and leave no sequelae. However, a small proportion of pediatric patients might present as having mild TBI but resulting in intracranial injuries that need to be identified rapidly and treated in a timely way. Neurological examination of infants younger than 2 years old is very difficult. Besides, concern of the parents for their children and fear of malpractice litigation may force the physicians to request radiological imaging, especially the CT scans. Although radiologic examination can effectively identify TBI, more selective use of CT in the youngest children would induce the number of children exposed to the risk of radiation-induced malignancy [3–5]. The aims of this study were to identify clinical predictors of intracranial injuries on CT in infants younger than 2 years old with mild TBI and identify clinical factors using radiologic examination.

## RESULTS

Between January 1, 2014, and December 30, 2016, 214 infants younger than 2 years old presented to Yijishan Hospital of Wannan Medical College with mild TBI. Patient demographics are presented in Table 1. Among them, 30 (5.8%) intracranial injuries occurred, 83 (16.1%) were girls, and the mean age was 14.7±7.2 months. Intracranial injuries on CT were observed in 3 of 7 (42.9%) of those younger than 3 months, 4 of 18 (22.2%) of those aged 3 to younger than 6 months, 8 of 37 (21.6%) of those aged 6 to younger than 12 months, and 15 of 152 (9.9%) of those aged 12 to 24 months. There was significant correlation between age in months and number of incidents (P=0.016). In 30 patients with intracranial injuries, the most common mechanism of injury was fall from elevation (n=26; 86.7%), and intracranial injuries were significantly correlated with mechanism of injury (P=0.0001). Compared with scalp hematoma group, the prevalence of intracranial injuries on CT was significantly lower in the patients without scalp hematoma (26/71, 36.6% vs. 4/143, 2.8%, P=0.0001).

**Table 1:** Characteristics of infants < 2 years old with mild traumatic brain injury

Characteristic	Intracranial injuries		Total	P Value
	Yes	No		
**Age (months)**				0.016
0–<3	3	4	7	
3–<6	4	14	18	
6–<12	8	29	37	
12–<24	15	137	152	
**Sex**				0.105
Female	16	67	83	
Male	14	117	131	
**Mechanism of injury**				0.0001
Fall from elevation	26	70	96	
Fall from standing/walking	2	79	81	
Traffic accidents	2	13	15	
Collision	0	12	12	
Object struck head–accidental	0	10	10	
**Scalp hematoma**				0.0001
Yes	26	45	71	
No	4	139	143	
**Total**	30	184	214	

In 71 infants with scalp hematomas, the age was classified into 4 groups (Table 2). The prevalence of intracranial injuries differed significantly between the groups and was higher in the younger age groups (P=0.003). In 26 scalp hematoma patients with intracranial injuries, the most common mechanism of injury was fall from elevation (n=23; 88.5%). The size of scalp hematomas was classified as large (>6 cm) in 15 (21.1%) infants, medium (>3,≦6 cm) in 29 (40.8%) infants, and small (≦3 cm) in 27 (38.0%) infants (Table 2). Intracranial injuries were significantly correlated with a larger scalp hematoma (P=0.0001). We assessed intracranial injuries according to the location of the scalp hematoma (frontal, temporal, parietal, occipital, ≧2 locations). There was a significant difference among the scalp hematoma locations (P=0.0001) (Table 2).

**Table 2:** Characteristics of 71 mild traumatic brain injury infants with scalp hematomas

Characteristic	Intracranial injuries		Total	P Value
	Yes	No		
**Age (months)**				0.003
0–<3	3	0	3	
3–<6	4	1	5	
6–<12	6	6	12	
12–<24	13	38	51	
**Sex**				0.205
Female	12	14	26	
Male	14	31	45	
**Mechanism of injury**				0.0001
Fall from elevation	23	8	31	
Fall from standing/walking	1	27	28	
Traffic accidents	2	2	4	
Collision	0	6	6	
Object struck head–accidental	0	2	2	
**Scalp hematoma**				
**Size (cm)**				0.0001
0-≦3	1	26	27	
3-≦6	13	16	29	
>6	12	3	15	
**Location**				0.0001
Frontal	4	20	24	
Temporal	1	1	2	
Parietal	8	10	18	
Occipital	2	12	14	
≧2 locations	11	2	13	
**Total**	26	45	71	

Multivariate logistic regression analysis revealed that independent predictors of intracranial injuries were scalp hematoma and mechanism of injury in infants younger than 2 years old with mild TBI (hazard ratio=38.291, P=0.0001; hazard ratio=0.174, P=0.001) (Table 3). In subgroup of mild TBI infants with scalp hematomas, multivariable analysis showed age, scalp hematoma size and mechanism of injury were each associated with intracranial injuries on CT, whereas scalp hematoma location was not (hazard ratio=0.299, P=0.032; hazard ratio=5.272, P=0.006; hazard ratio=0.312, P=0.030). The prognosis of all injured infants was good.

**Table 3:** Logistic regression analysis for independent factors

214 infants < 2 years old with mild traumatic brain injury			
**Factor**	**HR**	**95% CI**	**P Value**
**Age**	0.518	0.264-1.018	0.056
**Mechanism of injury**	0.174	0.060-0.506	0.001
**Scalp hematoma**	38.291	10.632-137.908	0.0001
**71 mild traumatic brain injury infants with scalp hematomas**			
**Factor**	**HR**	**95% CI**	**P Value**
**Age**	0.299	0.099-0.900	0.032
**Mechanism of injury**	0.312	0.109-0.894	0.030
**Scalp hematoma location**	1.602	0.863-2.973	0.135
**Scalp hematoma size**	5.272	1.627-17.078	0.006

HR, hazard ratio; CI, confidence interval.

## DISCUSSION

Children patients are particularly susceptible to TBI because of their anatomy [6]. Children's heads are disproportionately larger than those of adults, and their skulls are more compliant. Children also have weaker neck muscles and, most importantly, their neurons are less myelinated. Therefore, the children brain is not only more likely to sustain shearing forces when a force is applied but also, because of the decreased myelination, is more prone to axonal damage [6, 7]. Because the central nervous system is immature, it has typically been considered to be more difficult to detect clinical signs of intracranial lesions in infants <2 years old [8]. As a result, the rates of CT use have increased rapidly in children, however, the relatively high radiation doses associated with CT have raised health concerns [4, 9]. Thus, in the present study, we focused on mild TBI and intracranial injuries on CT in infants ranging in age from birth to 2 years old.

Similar to previous studies, infants younger than 3 months had a substantially higher prevalence of intracranial injuries on CT than infants in older age groups, and the age remained a substantial risk factor for intracranial injury in infants <2 years old [10, 11]. For infants aged 3 to 12 months, the incidence of intracranial injuries was 21.8% (12/55), emphasizing the importance of maintaining a low threshold for obtaining skull radiographs or cranial CT scans if there is a concern for intracranial injuries.

Falls are a common cause of traumatic injuries in children, and we have shown that intracranial injuries differ significantly between fall injury patterns. The most common mechanism of intracranial injuries in children with mild head trauma was a fall from elevation. Previous studies including children <5 years old reported the highest number of falls from a window, balcony, stairs, and furniture, and intracranial injury was a major cause of fall-related deaths in children [12, 13]. Our results suggest that parents should always be careful when there is a risk for fall from elevation.

A previous investigation reported that subcutaneous hematomas were observed in 28.7% of infants <24 months old, and a correlation with TBI was noted [14]. In the present study, subcutaneous hematomas were observed in 33.2% (71/214) of patients. We found that the scalp hematoma is a useful predictor of intracranial injuries in infants <2 years old with mild TBI. Furthermore, we investigated the characteristics of 71 mild TBI infants with scalp hematomas and revealed a close relationship between the size and location of the scalp hematoma and intracranial injuries in infants younger than 24 months with mild TBI. Scalp hematomas >3 cm in size might be determined to be a risk factor for intracranial injuries. The hematomas ≧2 locations was associated with a very high risk of intracranial injuries.

In accordance with previous investigations, the scalp hematoma and mechanism of injury were independent predictors of intracranial injuries on CT in our study [14, 15]. The literature indicates that a severe mechanism independently increases the risk of intracranial injuries, and falls from greater heights increase the prevalence of skull fractures traumatic brain injury [16], which suggests that clinicians should closely observe children with scalp hematomas after more severe mechanisms of injury in the emergency department. The multivariate logistic regression analysis in the subgroup of 71 infants with scalp hematomas also revealed that the age, mechanism of injury and scalp hematoma size were independent prognosticators of intracranial injuries. This suggests that the younger infants with larger scalp hematomas are more prone to suffer intracranial injuries.

The available research literature indicates that few pediatric patients with mild TBI require surgical intervention (0-1%) [8]. In our series, all injured infants had a good outcome. Although CT scan can identify intracranial injuries with high-level sensitivity, we should carefully select candidates for CT, the effects of radiation exposure must be considered. Use of CT scans in children to deliver cumulative doses of about 50 mGy might almost triple the risk of leukaemia and doses of about 60 mGy might triple the risk of brain cancer [3]. According to another study, infants <2 years old undergoing cranial CT had a higher risk of developing cancer compared with older children [17]. Otherwise, unnecessary tests with high costs and wasted time would also result. The results of this study are useful in determining whether to perform CT for infants with mild TBI.

The present study has some limitations. Clinicians obtained CT scans for the minority of patients, with selection bias likely toward those with more severe findings. Because this bias would be expected to inflate the prevalence of intracranial injuries on CT, the actual prevalence of intracranial injuries in infants with scalp hematomas is likely lower than that reported here.

## CONCLUSION

We found that the age, mechanism of injury and size of scalp hematomas were independently correlated with intracranial injuries on CT in TBI infants <2 years old with scalp hematoma. These factors should be considered as indications for radiologic examination in this subgroup.

## MATERIALS AND METHODS

This retrospective study was approved by the Institutional Ethical Board of the Yijishan Hospital of Wannan Medical College. All medical records of infants younger than 2 years old who had been hospitalized for mild TBI between January 1, 2014, and December 30, 2016, were reviewed. Data collected included sex, age, mechanism of head injury, scalp hematoma size, scalp hematoma location, neurologic examination and CT presentation.

Frequencies and descriptive statistics were used to characterize the study population. Either x^2^ test or Fisher exact test was used to determine differences between large and small scalp hematomas. In multivariable logistic regression analysis, we assessed the association between intracranial injuries with age, sex, scalp hematoma size and location.
